# Bone complications in patients with multiple myeloma in five European countries: a retrospective patient chart review

**DOI:** 10.1186/s12885-020-6596-y

**Published:** 2020-03-03

**Authors:** María-Victoria Mateos, Leah Fink, Niranchana Koneswaran, Michele Intorcia, Christina Giannopoulou, Daniela Niepel, Michele Cavo

**Affiliations:** 1 Haematology Service, University Hospital Salamanca/IBSAL, Paseo San Vicente 58–182, 37007 Salamanca, Spain; 2 Kantar Health, Paris, France; 3 Amgen (Europe) GmbH, Rotkreuz, Switzerland; 4 Amgen (Global Medical Affairs) GmbH, Rotkreuz, Switzerland; 5 Seragnoli Institute of Haematology and Medical Oncology, Bologna University School of Medicine, Bologna, Italy

**Keywords:** Chart review, Bisphosphonates, Bone complications, Denosumab, Europe, EU5, Multiple myeloma, Renal impairment, Skeletal-related events, Zoledronic acid

## Abstract

**Background:**

Bone complications (pathologic fracture, spinal cord compression, surgery to bone and radiation to bone) are a common problem in patients with multiple myeloma (MM). We set out to provide insights into the real-world burden of bone complications in patients with newly diagnosed MM (NDMM).

**Methods:**

We conducted a retrospective review of medical charts of patients with NDMM whose disease had progressed following first-line treatment in the 3 months before data collection in 2016 in five European countries (France, Germany, Italy, Spain and the United Kingdom).

**Results:**

The aggregated study population included 813 patients. Bone pain commonly led to MM diagnosis (63%) and 74% of all patients had two or more bone lesions at initiation of first-line treatment. Furthermore, 26% of patients experienced a new bone complication between MM diagnosis and disease progression following first-line treatment, despite 75% of individuals receiving bisphosphonates. Most bone complications (52%) occurred in the period before initiation of first-line treatment (mean duration: 2.3 months) and more than half of patients (56%) who experienced a new bone complication were hospitalised. Analgesics were used more frequently in patients with bone complications than in those without them (76% vs 50%, respectively). Furthermore, 51% of patients had renal impairment by the time first-line treatment was started. Overall, 25% of patients did not receive bisphosphonates for prevention of bone complications and one in four of those with renal impairment at initiation of first-line treatment did not receive bisphosphonates.

**Conclusions:**

Bone complications are common in patients with NDMM. They are frequently associated with hospitalization and analgesic use. Data from this study, conducted in the era of novel anti-myeloma therapies and before the approval of denosumab for use in patients with MM, suggest that although most patients (75%) received bisphosphonates, use of anti-resorptive therapy for prevention of bone complications may be suboptimal in patients with NDMM, irrespective of renal function.

## Background

Multiple myeloma (MM) is an incurable but treatable malignancy involving uncontrolled proliferation of malignant plasma cells in bone marrow [1]. Lytic bone lesions are an important feature of MM [1–4]. They are present in up to 80% of patients at diagnosis [3], and place a substantial additional burden on patients and healthcare systems [5, 6]; in particular, they cause pain and hypercalcemia, and lead to bone complications [7, 8]. Bone complications, also referred to as skeletal-related events, include pathologic fracture (PF), spinal cord compression (SCC), radiation to bone (RB) and surgery to bone (SB) [9, 10]. They may lead to increased mortality in patients with MM [11–13], and contribute towards disability and reduced health-related quality of life [5, 14], although individuals with bone complications may have more advanced disease [15, 16]. Management of bone complications is also associated with considerable healthcare resource use [6, 17–19]. Although outcomes for patients with MM continue to improve owing to the expanding number of effective treatments available [20, 21], bone complications remain a significant problem [8].

As bone complications place a substantial burden on patients and healthcare systems, their prevention is important [7, 22]. This can be achieved using anti-resorptive agents, including bisphosphonates or the receptor activator of nuclear factor kappa-B ligand (RANKL) inhibitor, denosumab [10, 23]. Both agents are approved for use in patients with MM in Europe [24–27]. Denosumab approval in this population was granted in 2018 based on the results of a large phase 3 randomized controlled trial that demonstrated its non-inferiority to zoledronic acid in delaying first on-study bone complication in patients with newly diagnosed MM (NDMM) [10, 27, 28]. Recommendations for bisphosphonate use are provided in clinical practice guidelines [3, 7, 29], and the more recent American Society of Clinical Oncology and National Comprehensive Cancer Network guidelines suggest denosumab as an alternative to bisphosphonates in patients with MM [30, 31]. Importantly, however, there is evidence to suggest that patients with lytic bone lesions from MM may receive suboptimal treatment for prevention of bone complications [32–34]. Suboptimal treatment can occur from multiple factors including late initiation, modified frequency of administration and length of therapy. Early initiation of treatment is of importance as many patients present with bone complications when diagnosed with MM. For example, in a recent phase III study of patients with newly diagnosed MM, 67% of patients enrolled in the study already presented with bone complications [10]. Despite the use of novel anti-myeloma agents and anti-resorptive agents, bone complications continue to occur within the first months of starting MM treatment [9, 10] and throughout the disease course, including in patients without a prior history of bone complications [16, 35].

Renal impairment (RI) is also an important feature of MM [1, 4, 36, 37]. Its severity is classified by the International Myeloma Working Group according to evidence of kidney damage and estimated glomerular filtration rate (eGFR) [36, 37]. Approximately 30–40% of individuals with MM have evidence of RI at diagnosis and 25–50% experience RI over the course of their disease [36]. RI at MM diagnosis is associated with significantly shorter overall survival and, although reversal of RI can improve survival, it remains reduced relative to that in patients without RI at diagnosis [38]. Bisphosphonates are nephrotoxic and require dose adjustment in patients with RI [7, 24–26], so concerns about renal function may lead to a decision not to treat with these agents [32]. Denosumab, unlike bisphosphonates, is not cleared by the kidneys and does not require dose adjustment in patients with RI [27].

Data from clinical trials may underestimate the real-world burden of bone complications because patients enrolled tend to be younger and fitter than those treated by physicians in routine clinical practice. Relatively few reported studies have investigated the real-world burden of bone complications in MM, despite the malignancy having one of the highest rates of bone involvement [13, 39]. Consequently, we conducted a large, retrospective patient chart review in five European countries in 2016 to understand how bone complications were being managed in individuals with symptomatic NDMM in the period just before the approval of denosumab for use in patients with MM. Data were collected on the frequency of bone complications and bone complication-related hospitalizations, bisphosphonate use and analgesic use in the period between MM diagnosis and disease progression following first-line treatment.

## Methods

### Study population

Patients with symptomatic NDMM from five European countries (EU5; France, Germany, Italy, Spain and the United Kingdom [UK]) whose disease had progressed following first-line treatment were included in the study. Physicians specialising in oncology, hematology and hemato-oncology, and internists (in Germany only), were eligible to contribute patient data to the study providing they were responsible for initiating anti-myeloma drug treatment, managed a minimum of 15 patients with MM per month (10 patients for office-based physicians in Germany) and had a minimum of 3 years of clinical experience. For the study to be representative of the real-world management of patients with MM, physicians were recruited in each country according to regional and practice setting quotas (hospital types, office-based for Germany). These quotas were set according to the distribution of physicians across each country.

### Study design

Data were extracted retrospectively from the medical records of patients onto anonymized study-specific case report forms (CRFs). This took place over a 2–4-week period between June and July 2016 in France, Italy and the UK, and between September and November 2016 in Germany and Spain. Each physician included eligible patients consecutively and in reverse chronological order. Patients were eligible for inclusion in the study if they had a diagnosis of symptomatic MM, were aged over 18 years at the time of data collection and had experienced disease progression following first-line treatment in the 3 months before data collection. Patients who participated in a clinical trial during first-line treatment were excluded.

### Study data

Data were reported in the overall period between MM diagnosis and disease progression following first-line treatment (the at-risk period) and at various stages in the treatment pathway: before the start of first-line induction therapy (period 1); during active first-line treatment (induction and maintenance therapy) (period 2); and after first-line treatment discontinuation (period 3). Data collected included patient and disease characteristics at diagnosis and at initiation of first-line treatment, and frequency of bone complications, frequency of hospitalizations due to bone complications, use of bisphosphonates and use of analgesic medications. Bone complications were defined as pathologic fracture (PF), spinal cord compression (SCC), radiation to bone (RB) and surgery to bone (SB). The CRF allowed a maximum of one bone complication of each type to be recorded for each patient in any given period. PF and SCC were recorded at diagnosis only if they led to the diagnosis, and the CRF did not allow RB and SB to be recorded at diagnosis. Bisphosphonate use was recorded in periods 2 and 3, and according to whether patients had RI at initiation of first-line treatment. Denosumab was not approved for use in patients with MM at the time of data collection; therefore, only information on bisphosphonate use was recorded on the CRF. RI was recorded at initiation of first-line treatment; severity of RI was defined as mild (creatinine clearance [CrCl] ≥ 50 mL/min), moderate (CrCl 30–49 mL/min) or severe (< 30 mL/min). Normal renal function was not formally defined on the CRF. RI was recorded at diagnosis only if it led to the diagnosis; it was recorded as physician-assessed “renal dysfunction” without further categorization by severity. Analgesic use was recorded in periods 2 and 3 according to whether patients had experienced bone complications and was defined using the three-step World Health Organization analgesic ladder [40].

### Data analysis

Data were reported for the aggregated EU5 and for the five individual European countries. To estimate the aggregated EU5 data, country-specific data were weighted based on the MM incidence in the respective countries. Weights were assigned as follows: France (27%), Germany (22%), Italy (21%), Spain (10%) and the UK (20%). No other weighting was applied to the data. Missing or incomplete data were marked as unavailable if they could not be captured after communication with the participating physician, and quality control checks were conducted at study completion and at the data analysis stage. Descriptive statistics were used to summarize the data and no formal hypothesis testing was performed.

## Results

### Sample characteristics

In total, 391 physicians participated in the study (France, 82; Germany, 91; Italy, 85; Spain, 75; UK, 58). Data were collected on 808 patients whose disease had progressed following first-line treatment (France, 146; Germany, 175; Italy, 173; Spain, 141; UK, 173) and, after weighting by MM incidence, 813 individuals comprised the aggregated EU5 study population. Overall, the mean cumulative time at risk of bone complications was 28.2 months (95% confidence interval [CI]: 26.6–29.9 months). The mean cumulative time at risk was 2.3 months (95% CI: 1.6–3.0 months) in period 1, 9.2 months (95% CI: 8.5–9.9 months) in period 2 and 16.5 months (95% CI: 15.1–17.9 months) in period 3. Patient and disease characteristics at diagnosis and at initiation of first-line treatment are shown in Table 1. The prevalence of bone lesions was not recorded at diagnosis; however, 74% of patients had at least two bone lesions at initiation of first-line treatment. Most patients (78%) had at least one of bone pain, hypercalcemia, SCC or PF leading to diagnosis; 63% had bone pain, 27% had PF, 21% had hypercalcemia and 4% had SCC.

**Table 1:** Characteristics of patients in the study population

Characteristic	EU5^a^ *N* = 813	France *N* = 146	Germany *N* = 175	Italy *N* = 173	Spain *N* = 141	UK *N* = 173
Median age at diagnosis, years (IQR)	66 (58–74)	65 (57–73)	68 (60–74)	65 (57–72)	66 (61–74)	65 (57–75)
Male, n (%)	477 (59)	83 (57)	106 (61)	99 (57)	85 (60)	103 (60)
SCT during total at-risk period, n (%)^b^	317 (39)	64 (44)	58 (33)	73 (42)	49 (35)	65 (38)
ISS stage at diagnosis, n (%)						
1	120 (15)	22 (15)	23 (13)	28 (16)	27 (19)	22 (13)
2	282 (35)	46 (32)	49 (28)	56 (32)	58 (41)	75 (43)
3	380 (47)	74 (51)	95 (54)	78 (45)	51 (36)	71 (41)
Unknown	31 (4)	3 (2)	8 (5)	11 (6)	5 (4)	5 (3)
ECOG performance status at 1 L initiation, n (%)						
0	138 (17)	11 (8)	34 (19)	43 (25)	19 (14)	36 (21)
1	467 (57)	86 (59)	110 (63)	84 (49)	77 (55)	103 (60)
2	181 (22)	44 (30)	27 (15)	43 (25)	37 (26)	26 (15)
≥ 3	20 (3)	5 (3)	3 (2)	3 (2)	7 (5)	2 (1)
Unknown	8 (1)	0	1 (1)	0	1 (1)	6 (4)
Circumstances that led to MM diagnosis, n (%)						
Bone pain	513 (63)	97 (66)	109 (62)	96 (56)	95 (67)	111 (64)
HC	174 (21)	30 (21)	29 (17)	30 (17)	30 (21)	52 (30)
VF	188 (23)	37 (25)	45 (26)	42 (24)	22 (16)	35 (20)
OF	34 (4)	4 (3)	6 (3)	13 (8)	6 (4)	6 (4)
SCC	29 (4)	5 (3)	4 (2)	8 (5)	4 (3)	7 (4)
≥ 1 bone-related problem^c^	634 (78)	117 (80)	135 (77)	128 (74)	110 (78)	137 (79)
≥ 1 HC, VF, OF or SCC	359 (44)	67 (46)	71 (41)	79 (46)	56 (40)	79 (46)
≥ 1 VF, OF or SCC	233 (29)	45 (31)	52 (30)	58 (34)	30 (21)	42 (24)
Renal impairment	187 (23)	29 (20)	55 (31)	25 (15)	26 (18)	49 (28)
Presentation at the initiation of 1 L, n (%)						
≥ 2 bone lesions	598 (74)	111 (76)	138 (79)	127 (73)	96 (68)	118 (68)

*1 L* first-line treatment, *ECOG* Eastern Cooperative Oncology Group, *EU5* five European countries (France, Germany, Italy, Spain and the UK), *HC* hypercalcemia, *IQR* interquartile range, *ISS* international staging system, *MM* multiple myeloma, *OF* other non-vertebral fracture, *SCC* spinal cord compression, *SCT* stem cell transplantation, *UK* United Kingdom, *VF* vertebral fracture

^a^Aggregated EU5 data have been weighted based on the MM incidence in each country so base sizes for individual countries may not equal the EU5 total

^b^Overall study period from MM diagnosis to disease progression following 1 L

^c^Any of bone pain, HC, VF, OF and SCC

### Frequency of bone complications

**Fig. 1:**
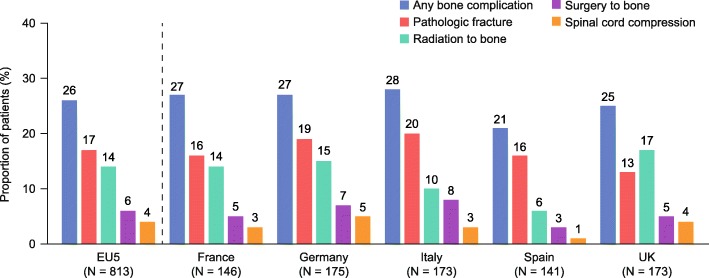
Proportion of patients who experienced bone complications The proportion of patients who experienced at least one bone complication (pathologic fracture, spinal cord compression, radiation to bone or surgery to bone) during the total at-risk period is expressed as a percentage of the total number of patients (from multiple myeloma diagnosis to disease progression following first-line treatment). Data are presented for the aggregated analysis across the EU5 and for individual countries. Aggregated EU5 data have been weighted based on the multiple myeloma incidence in each country so base sizes for individual countries may not equal the EU5 total. *EU5* five European countries (France, Germany, Italy, Spain and the UK), *UK* United Kingdom

Overall, 366 bone complications (PF, SCC, RB or SB) were recorded across all three at-risk periods between MM diagnosis and disease progression following first-line treatment. At least one bone complication was experienced by 214 patients (26%; with a mean incidence of 1.5 [95% CI: 1.4–1.6]) (Table 2, Fig. 1). PF was the most common finding; 64% of patients who experienced at least one bone complication had a PF, compared with 51% for RB, 22% for SB and 14% for SCC (Table 2). Patients who experienced a bone complication (PF, SCC, RB or SB) during the total at-risk period were more likely to have at least one of bone pain, hypercalcemia, PF or SCC leading to diagnosis than those who did not (90% vs 73%, respectively). They were also more likely to have PF or SCC leading to diagnosis than those without a bone complication (56 and 19%, respectively).

**Table 2:** Frequency of bone complications

Outcome	At-risk period^a^	EU5^b^ *N* = 813	France *N* = 146	Germany *N* = 175	Italy *N* = 173	Spain *N* = 141	UK *N* = 173
Patients who experienced at least one bone complication, n (%)^c^							
Any bone complication	Overall	214 (26)	40 (27)	48 (27)	49 (28)	29 (21)	43 (25)
	Period 1	135 (17)	24 (16)	28 (16)	36 (21)	19 (14)	26 (15)
	Period 2	67 (8)	12 (8)	18 (10)	10 (6)	5 (4)	18 (10)
	Period 3	72 (9)	17 (12)	17 (10)	17 (10)	9 (6)	9 (5)
Mean number of bone complications per patient who experienced at least one bone complication, mean (95% CI)							
Any bone complication	Overall	1.51 (1.41–1.61)	1.43 (1.26–1.60)	1.69 (1.45–1.93)	1.47 (1.27–1.67)	1.28 (1.00–1.56)	1.56 (1.29–1.83)
	Period 1	1.43 (1.32–1.55)	1.46 (1.22–1.70)	1.56 (1.28–1.83)	1.33 (1.12–1.55)	1.18 (0.95–1.41)	1.54 (1.26–1.81)
	Period 2	1.27 (1.11–1.43)	1.21 (0.93–1.49)	1.12 (0.96–1.27)	1.33 (1.03–1.64)	1.33 (0.80–1.87)	1.42 (1.00–1.84)
	Period 3	1.25 (1.12–1.38)	1.19 (1.04–1.34)	1.31 (0.94–1.69)	1.31 (1.03–1.60)	1.40 (0.97–1.83)	1.20 (0.95–1.45)
Patients who experienced at least one bone complication by type of bone complication and at-risk period, n (%)^d^							
PF	Overall	137 (64)	23 (58)	34 (71)	35 (71)	23 (79)	23 (54)
	Period 1	99 (74)	16 (67)	22 (79)	29 (81)	18 (95)	16 (62)
	Period 2	19 (28)	4 (33)	3 (17)	4 (40)	1 (20)	5 (28)
	Period 3	40 (56)	8 (47)	12 (71)	10 (59)	6 (67)	4 (44)
SCC	Overall	29 (14)	5 (13)	8 (17)	6 (12)	2 (7)	7 (16)
	Period 1	21 (15)	5 (21)	3 (11)	4 (11)	2 (11)	5 (19)
	Period 2	5 (8)	0	2 (11)	0	0	3 (17)
	Period 3	9 (13)	1 (6)	3 (18)	3 (18)	0	2 (22)
RB	Overall	110 (51)	21 (53)	27 (56)	18 (37)	8 (28)	29 (67)
	Period 1	47 (35)	11 (46)	10 (36)	7 (19)	0	13 (50)
	Period 2	44 (65)	6 (50)	11 (61)	6 (60)	4 (80)	15 (83)
	Period 3	31 (42)	8 (47)	6 (35)	5 (29)	5 (56)	5 (56)
SB	Overall	46 (22)	8 (20)	12 (25)	13 (27)	4 (14)	8 (19)
	Period 1	26 (19)	2 (8)	8 (29)	8 (22)	2 (11)	6 (23)
	Period 2	17 (25)	4 (33)	4 (22)	3 (30)	2 (40)	3 (17)
	Period 3	10 (13)	3 (18)	1 (6)	4 (24)	1 (11)	0

*CI* confidence interval, *EU5* five European countries (France, Germany, Italy, Spain and the UK), *PF* pathologic fracture, *RB* radiation to bone, *SB* surgery to bone, *SCC* spinal cord compression, *UK* United Kingdom

^a^Overall: from multiple myeloma diagnosis to disease progression following first-line treatment; period 1: before initiation of induction therapy; period 2: during active first-line therapy; period 3: after treatment discontinuation

^b^Aggregated EU5 data have been weighted based on the multiple myeloma incidence in each country so base sizes for individual countries may not equal the EU5 total

^c^Expressed as a percentage of the total number of patients

^d^Expressed as a percentage of the number of patients who experienced at least one bone complication

By at-risk period, 192 bone complications (52%) were recorded during period 1, 84 (23%) in period 2 and 90 (25%) in period 3. A greater proportion of patients experienced at least one bone complication during period 1 (17%) than in period 2 (8%) or period 3 (9%) (Table 2); however, some bone complications recorded before initiation of first-line treatment (period 1) may already have been present at diagnosis. The mean incidence of bone complications for patients who experienced at least one was similar across all periods (1.4 [95% CI: 1.3–1.6] in period 1 and 1.3 [95% CI: 1.1–1.4] in both periods 2 and 3; Table 2). During the total at-risk period, PF was the most common bone complication in period 1 (74%) and period 3 (56%); however, 65% of patients who experienced at least one bone complication during period 2 required RB (Table 2).

### Hospitalizations due to bone complications

**Fig. 2:**
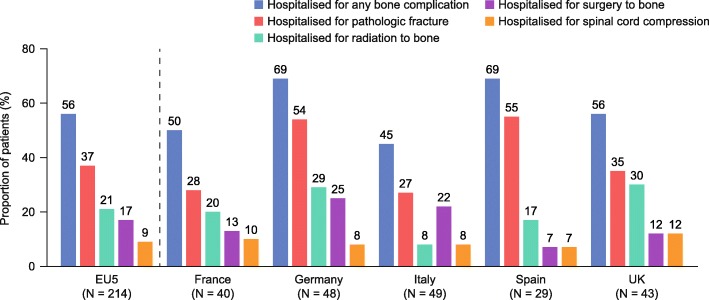
Proportion of patients requiring bone complication-related hospitalization The proportion of patients who were hospitalised is expressed as a percentage of the total number of patients who experienced a bone complication (pathologic fracture, spinal cord compression, radiation to bone or surgery to bone) during the total at-risk period (from multiple myeloma diagnosis to disease progression following first-line treatment). Data are presented across the EU5 and for individual countries. Aggregated EU5 data have been weighted based on the multiple myeloma incidence in each country so base sizes for individual countries may not equal the EU5 total. *EU5* five European countries (France, Germany, Italy, Spain and the UK); *UK* United Kingdom

Overall, 119 patients (15%) experienced 206 bone complications that resulted in hospitalization (Table 3). Of the individuals who experienced at least one bone complication, 56% were hospitalized (Fig. 2); 56% of all PF cases led to hospitalization, compared with 74% for SB, 69% for SCC and 44% for RB. By at-risk period, a greater proportion of patients were hospitalized during period 1 (9%) than in period 2 (4%) or period 3 (5%) (Table 3). During the total at-risk period, PF was the bone complication most commonly resulting in hospitalization in period 1 (74%) and period 3 (58%); however, 55% of patients hospitalized owing to bone complications during period 2 required RB (Table 3).

**Table 3:** Frequency of hospitalizations due to bone complications

Outcome	At-risk period^a^	EU5^b^ *N* = 813	France *N* = 146	Germany *N* = 175	Italy *N* = 173	Spain *N* = 141	UK *N* = 173
Hospitalizations due to bone complications by at-risk period, n (%)^c^							
Any bone complication	Overall	119 (15)	20 (14)	33 (19)	22 (13)	20 (14)	24 (14)
	Period 1	73 (9)	11 (8)	21 (12)	14 (8)	14 (10)	14 (8)
	Period 2	29 (4)	4 (3)	10 (6)	4 (2)	3 (2)	7 (4)
	Period 3	41 (5)	9 (6)	11 (6)	6 (4)	7 (5)	7 (4)
Hospitalizations due to bone complications by type of bone complication and at-risk period, n (%)^d^							
PF	Overall	79 (66)	11 (55)	26 (79)	13 (59)	16 (80)	15 (63)
	Period 1	54 (74)	6 (55)	19 (91)	8 (57)	13 (93)	11 (79)
	Period 2	11 (37)	1 (25)	3 (30)	2 (50)	0	4 (57)
	Period 3	24 (58)	5 (56)	8 (72)	3 (50)	5 (71)	3 (43)
SCC	Overall	20 (17)	4 (20)	4 (12)	4 (18)	2 (10)	5 (21)
	Period 1	15 (21)	4 (36)	2 (10)	2 (14)	2 (14)	4 (29)
	Period 2	2 (8)	0	0	0	0	2 (29)
	Period 3	7 (18)	1 (11)	2 (18)	2 (33)	0	2 (29)
RB	Overall	46 (39)	8 (40)	14 (42)	4 (18)	5 (25)	13 (54)
	Period 1	21 (28)	5 (46)	4 (19)	3 (21)	0	6 (43)
	Period 2	16 (55)	2 (50)	5 (50)	1 (25)	3 (100)	5 (71)
	Period 3	17 (41)	4 (44)	5 (46)	0	3 (43)	4 (57)
SB	Overall	36 (30)	5 (25)	12 (36)	11 (50)	2 (10)	5 (21)
	Period 1	20 (28)	1 (9)	8 (38)	7 (50)	1 (7)	4 (29)
	Period 2	10 (35)	1 (25)	4 (40)	2 (50)	1 (33)	2 (29)
	Period 3	9 (21)	3 (33)	1 (9)	3 (50)	1 (14)	0

*EU5* five European countries (France, Germany, Italy, Spain and the UK), *PF* pathologic fracture, *RB* radiation to bone, *SB* surgery to bone, *SCC* spinal cord compression, *UK* United Kingdom

^a^Overall: from multiple myeloma diagnosis to disease progression following first-line treatment; period 1: before initiation of induction therapy; period 2: during active first-line therapy; period 3: after treatment discontinuation

^b^Aggregated EU5 data have been weighted based on the multiple myeloma incidence in each country so base sizes for individual countries may not equal the EU5 total

^c^Expressed as a percentage of the total number of patients

^d^Expressed as a percentage of the number of patients who were hospitalized owing to a bone complication

### Bisphosphonate administration

Overall, 612 patients (75%) were treated with bisphosphonates and 88% of them received zoledronic acid. By at-risk period, 588 patients (72%) were treated with bisphosphonates during first-line induction, and 377 individuals (46%) were given bisphosphonates during period 3. The mean number of bisphosphonate doses received among treated patients was 15.4 (95% CI: 14.3–16.5) over a mean follow-up of 30 months (95% CI: 28–32 months).

### RI and bisphosphonate administration

**Fig. 3:**
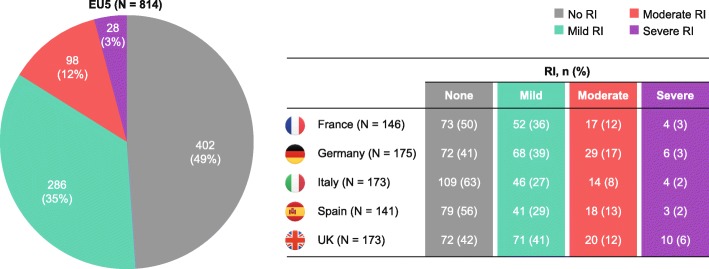
Renal function at initiation of 1 L treatment The proportion of patients with RI at initiation of 1 L treatment. Mild RI: CrCl ≥50 mL/min; moderate RI: CrCl 30–49 mL/min; severe RI: CrCl < 30 mL/min. Data are presented for the aggregated analysis across the EU5 and for individual countries. Aggregated EU5 data have been weighted based on the multiple myeloma incidence in each country so base sizes for individual countries may not equal the EU5 total. *1 L*, first line, *CrCl* creatinine clearance, *EU5* five European countries (France, Germany, Italy, Spain and the UK), *RI* renal impairment, *UK* United Kingdom

**Fig. 4:**
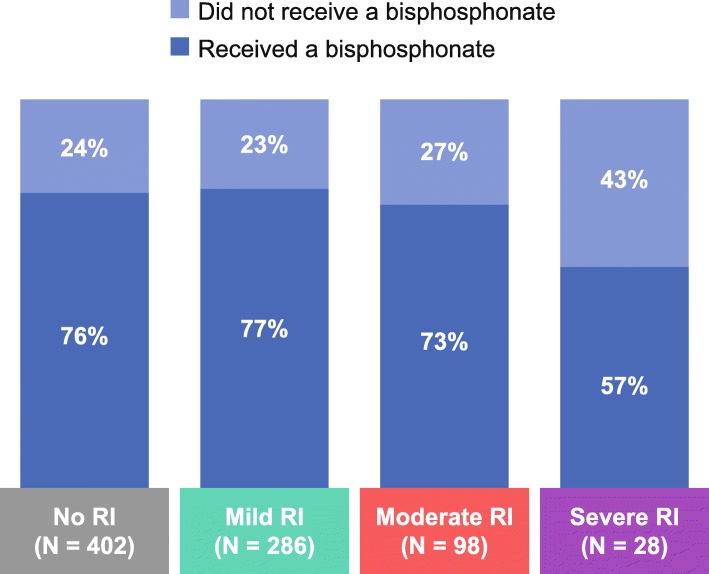
Bisphosphonate use according to renal function at initiation of 1 L treatment The proportion of patients who received treatment with bisphosphonates according to RI severity at initiation of 1 L treatment. Mild RI: CrCl ≥50 mL/min; moderate RI: CrCl 30–49 mL/min; severe RI: CrCl < 30 mL/min. Data are presented for the aggregated analysis across the EU5. Aggregated EU5 data have been weighted based on the multiple myeloma incidence in each country so base sizes for individual countries may not equal the EU5 total. *CrCl* creatinine clearance, *EU5* five European countries (France, Germany, Italy, Spain and the United Kingdom), *1 L* first line, *RI* renal impairment

Overall, 187 patients (23%) had RI leading to diagnosis and 412 (51%) had RI by the time first-line treatment was initiated. By severity, 286 patients (35%) had mild RI, 98 (12%) had moderate RI and 28 (3%) had severe RI at initiation of first-line treatment (Fig. 3). Overall, the proportions of patients treated with bisphosphonates were similar for individuals with (75%) and without (76%) RI; 77% of patients with mild RI, 73% with moderate RI and 57% with severe RI received bisphosphonates (Fig. 4). In general, patients with RI received reduced bisphosphonate doses relative to those without RI (data not shown).

### Analgesic administration

Overall, 57% of patients received analgesic medication, including 76% with bone complications and 50% without them (Table 4). Patients who experienced bone complications also received higher step analgesics than those with no bone complications (34% vs 20%, respectively, for step 2; 34% vs 15%, respectively, for step 3; Table 4). Fewer patients with bone complications were given analgesics in period 3 (42%) than in period 2 (75%) (Table 4). The proportion of patients with bone complications who received higher step analgesics was also lower in period 3 than in period 2 (15% vs 29%, respectively for step 2; 16% vs 28%, respectively for step 3). More patients with bone complications received analgesia, and higher step analgesia, in all at-risk periods than those without bone complications (Table 4).

**Table 4:** Analgesic use according to the experience of bone complications

Outcome	At-risk period^a^	Patient population	EU5^b^ *N* = 813	France *N* = 146	Germany *N* = 175	Italy *N* = 173	Spain *N* = 141	UK *N* = 173
Patients who received analgesics by at-risk period, n (%)^c^								
Any analgesic use	Overall	All patients	461 (57)	102 (70)	110 (63)	84 (49)	94 (67)	66 (38)
		With BCs	162 (76)	36 (90)	34 (71)	35 (71)	25 (86)	27 (63)
		Without BCs	299 (50)	66 (62)	76 (60)	49 (40)	69 (62)	39 (30)
	Period 2	All patients	457 (56)	104 (71)	110 (63)	84 (49)	93 (66)	60 (35)
		With BCs	160 (75)	34 (85)	35 (73)	33 (67)	26 (90)	28 (65)
		Without BCs	296 (49)	70 (66)	75 (59)	51 (41)	67 (60)	32 (25)
	Period 3	All patients	234 (29)	43 (30)	66 (38)	46 (27)	10 (7)	32 (19)
		With BCs	89 (42)	15 (38)	21 (44)	22 (45)	21 (72)	13 (30)
		Without BCs	145 (24)	28 (26)	45 (35)	24 (19)	31 (28)	19 (15)
Patients who received analgesics by step^c^ and at-risk period, n (%)^d^								
Step 1	Overall	All patients	255 (31)	38 (26)	70 (40)	56 (32)	51 (36)	47 (27)
Step 2			192 (24)	46 (32)	50 (29)	30 (17)	36 (26)	25 (15)
Step 3			162 (20)	51 (35)	37 (21)	21 (12)	34 (24)	10 (6)
Step 1		With BCs	73 (34)	11 (28)	18 (38)	21 (43)	9 (31)	14 (33)
Step 2			72 (34)	15 (38)	11 (23)	16 (33)	12 (41)	16 (37)
Step 3			72 (34)	21 (53)	18 (38)	10 (20)	12 (41)	7 (16)
Step 1		Without BCs	182 (30)	27 (26)	52 (41)	35 (28)	42 (38)	33 (25)
Step 2			120 (20)	31 (29)	39 (31)	14 (11)	24 (21)	9 (7)
Step 3			89 (15)	30 (28)	19 (15)	11 (9)	22 (20)	3 (2)
Step 1	Period 2	All patients	216 (27)	30 (21)	63 (36)	50 (29)	36 (26)	41 (24)
Step 2			165 (20)	39 (27)	49 (28)	23 (13)	31 (22)	19 (11)
Step 3			144 (18)	46 (32)	33 (19)	18 (10)	32 (23)	8 (5)
Step 1		With BCs	65 (30)	8 (20)	16 (33)	18 (37)	8 (28)	16 (37)
Step 2			62 (29)	14 (35)	12 (25)	12 (24)	9 (31)	13 (30)
Step 3			59 (28)	16 (40)	16 (33)	7 (14)	11 (38)	6 (14)
Step 1		Without BCs	150 (25)	22 (21)	47 (37)	32 (26)	28 (25)	25 (19)
Step 2			102 (17)	25 (24)	37 (29)	11 (9)	22 (20)	6 (5)
Step 3			86 (14)	30 (28)	17 (13)	11 (9)	21 (19)	2 (2)
Step 1	Period 3	All patients	130 (16)	17 (12)	39 (22)	30 (17)	52 (37)	21 (12)
Step 2			82 (10)	14 (10)	27 (15)	15 (9)	30 (21)	13 (8)
Step 3			56 (7)	15 (10)	17 (10)	7 (4)	13 (9)	5 (3)
Step 1		With BCs	40 (19)	5 (13)	9 (19)	15 (31)	7 (24)	6 (14)
Step 2			33 (15)	3 (8)	8 (17)	8 (16)	8 (28)	8 (19)
Step 3			33 (16)	3 (8)	8 (17)	4 (8)	7 (24)	4 (9)
Step 1		Without BCs	90 (15)	12 (11)	30 (24)	15 (12)	23 (21)	15 (12)
Step 2			49 (8)	11 (10)	19 (15)	7 (6)	5 (5)	5 (4)
Step 3			23 (4)	6 (6)	9 (7)	3 (2)	3 (3)	1 (1)

*BCs* bone complications, *EU5* five European countries (France, Germany, Italy, Spain and the UK), *UK* United Kingdom

^a^Overall: from multiple myeloma diagnosis to disease progression following first-line treatment; period 2: during active first-line therapy; period 3: after treatment discontinuation. Note that analgesic use was not recorded for period 1 (before initiation of induction therapy)

^b^Aggregated EU5 data have been weighted based on the multiple myeloma incidence in each country so base sizes for individual countries may not equal the EU5 total

^c^Step on the World Health Organization analgesic ladder

^d^Expressed as a percentage of the total number of patients, patients with BCs or patients without BCs, as indicated (patient numbers by country – EU5 with BCs: 214; EU5 without BCs: 599; France with BCs: 40; France without BCs: 106; Germany with BCs: 48; Germany without BCs: 127; Italy with BCs: 49; Italy without BCs: 124; Spain with BCs: 29; Spain without BCs: 112; UK with BCs: 43; UK without BCs: 130)

## Discussion

We conducted a large multicenter European chart review that assessed the real-world burden of bone complications on patients with NDMM in the era of novel anti-myeloma therapies and before the approval of denosumab for use in patients with MM [20, 21, 27]. The results of our analysis showed that most patients had evidence of bone lesions at initiation of first-line treatment. Bone pain commonly led to diagnosis (63%), and hypercalcemia and PF led to diagnosis in approximately one-quarter of cases. These results are consistent with those of an earlier retrospective European chart review conducted in 2014 by Yong et al. (*N* = 4997), in which patients had a history of bone pain (61%), hypercalcemia (19%), PF (30%) and SCC (1%) as a circumstance of diagnosis [41].

New bone complications were common in our study population, despite 75% of patients receiving bisphosphonate treatment. These results support the findings of other real-world studies in patients with MM. For example, a retrospective analysis of United States (US) claims data by Nash Smyth et al. (*N* = 1028) found that 58% of patients with NDMM experienced at least one bone complication [17]. It should be noted, however, that the definition of bone complications in that study included hypercalcemia and the mean follow-up was 21.4 months. Our data also suggest that patients with MM are at risk of multiple bone complications and that the risk is higher in those with prior bone complications. Consistent with our findings, Nash Smyth et al. estimated that, among patients who experienced at least one bone complication, 50% had one, 26% had two and 24% had at least three bone complications [17]. Furthermore, a retrospective US database analysis by Kim et al. (*N* = 343) found that the incidence of bone complications 1 year after MM diagnosis for patients with a prior history of bone complications was 103 per 100 person-years compared with 16 per 100 person-years for individuals with no prior history [16]. The incidence of bone lesions was not recorded throughout our study, so it was not possible to establish the extent to which new bone complications were associated with changes in the burden of myeloma bone disease.

We also found that approximately half of bone complications occurred before initiation of first-line treatment (over a mean duration of 2.3 months); however, some of these may have already been present at diagnosis. Results from other studies suggest that most bone complications occur within the first year after MM diagnosis [10, 16]; for example, Kim et al. reported that 68% of bone complications occurred during this period [16]. In a recent randomized controlled trial in patients with NDMM (*N* = 1718), 60% of first on-study bone complications were experienced during the initial 3 months and 81% during the initial 6 months of the study [10].

Our analysis showed that bone complications were often associated with hospitalization, with most cases of SB and SCC, and around half the cases of PF and RB, resulting in this outcome. Analysis of data from a retrospective European chart review of patients with bone metastases or bone lesions from MM (*N* = 631) found that 31–36% of bone complications required hospitalization [42]. As in our study, SB and SCC were associated with higher rates of inpatient stays than other bone complications [42].

Bone complications can be associated with substantial pain in patients with MM [5, 8]. In our study, individuals with bone complications required more frequent and stronger analgesic medication than those without them, and 57% of patients overall required analgesia. This proportion is similar to that reported by Yong et al., who found that 21, 16 and 13% of patients received step 1, 2 and 3 analgesia, respectively, at first-line treatment [41]. Importantly, bone pain can be debilitating and is associated with reduced health-related quality of life [5, 14].

Most patients in our study received bisphosphonates, although there was wide variation in their use between individual countries, most likely reflecting heterogeneous clinical practice across European healthcare systems [32]. Zoledronic acid was the most widely used bisphosphonate, which is consistent with findings of other studies [32, 34]. Although most patients received bisphosphonates in our study, a substantial proportion (25%) were untreated. There may be many reasons why patients do not receive bisphosphonates, including contraindications, tolerability or the absence of bone lesions, however, as our analyses were retrospective in nature, we did not assess this in our study. Overall, our results are consistent with data from other observational studies suggesting that anti-resorptive agent use may be suboptimal in patients with MM [33, 34, 43]. Analysis of data from a large retrospective US study by Kim et al. (*N* = 11,112; median follow-up: 22.6 months) found that 42% of patients did not start bisphosphonate treatment within a year after MM diagnosis [33]. Another retrospective study (*N* = 1309) found that, contrary to guideline recommendations, 45% of patients with NDMM did not receive bisphosphonate treatment within 6 months after starting anti-MM therapy [43]. Qian et al. (*N* = 9617) found that only 38.8% of patients with NDMM received bisphosphonates, and that these individuals had poor adherence to and persistence with treatment [34].

Early initiation of anti-resorptive treatment is important [44]; however, these therapies tend to be underused during the period between diagnosis and the start of first-line treatment [33, 34]. In patients with newly diagnosed bone metastases from solid tumors, Intorcia et al. showed that early initiation of anti-resorptive therapy (≤ 3 months after diagnosis) was associated with longer times to first and subsequent bone complications than late initiation (> 3–9 months after diagnosis) [45]. Kim et al. found that only 13% of patients received bisphosphonate therapy before initiation of first-line treatment [33]. As anti-resorptive treatment cannot start until a diagnosis of MM has been confirmed, diagnostic delay may result in the development of new bone complications in untreated patients. Additionally, fewer than half of patients in our study received bisphosphonates following first-line treatment despite European Myeloma Network guidelines recommending that individuals with bone lesions at diagnosis should be treated continuously with zoledronic acid [7], although most patients were given therapy during first-line induction. Consistent with our findings, Kim et al. also found evidence of anti-resorptive agent underuse during these at-risk periods; 52% of patients received concomitant bisphosphonates during first-line therapy [33]. This decreased to only 18% in the period between first-line and second-line treatment [33]. Furthermore, in the study by Yong et al., which was conducted in 2014 in the same European countries as our study (with the addition of Belgium and Switzerland), 66% of patients received bisphosphonates at first line (*N* = 1802 at first line) [41]. A substantial proportion of patients who experienced a new bone complication in our study did so while receiving anti-myeloma treatment and in the period afterwards. These data suggest that anti-resorptive agents are also likely to benefit patients during these periods in the disease course.

RI was common in our study population. Approximately half of patients had RI by the time first-line treatment was initiated, which is supportive of other real-world RI data in patients with NDMM [41, 46]. In a large retrospective US study including 8767 newly diagnosed patients (median follow-up: 14.3 months), the prevalence of RI (defined as at least one recorded eGFR < 60 mL/min/1.73 m^2^) and chronic kidney disease (CKD; defined as at least two records of eGFR < 60 mL/min/1.73 m^2^ ≥ 90 days apart) was 61 and 50%, respectively [46]. Associations between RI and bone complications have been reported, and RI has important implications for subsequent MM treatment [7, 17, 37, 41].

In our study, despite product label recommendations to the contrary [24, 25], 57% of patients with severe RI (CrCl < 30 mL/min) were treated with bisphosphonates. In one real-world study, at least 40% of patients with RI (*N* = 5334) or CKD (*N* = 3399) received nephrotoxic drugs, mostly bisphosphonates [46]. Furthermore, approximately one-quarter of patients with mild or moderate RI in our study did not receive bisphosphonates. Data from the study by Kim et al. suggested that poor renal function at baseline was associated with less frequent bisphosphonate treatment (CKD stage 5 vs stage 1: 24% vs 72%) and delays in starting bisphosphonate treatment (CKD stage 5 vs stage 1: median 70 vs 25 days from MM diagnosis) [33]. Results of subsequent retrospective database analyses have indicated that RI is associated with a decreased likelihood of bisphosphonate use and an increased likelihood of treatment interruption [43, 47]. Furthermore, findings from a physician survey suggested that some patients would never receive bisphosphonates because of “renal issues” [32]. Our study, together with these published data, highlights the lack of treatment options available for the prevention of bone complications in patients with MM in the era before denosumab.

The RANKL inhibitor denosumab is the latest anti-resorptive agent to be approved in Europe for use in patients with MM [27]. In a large phase 3 randomized controlled trial, denosumab demonstrated non-inferiority to zoledronic acid in delaying first on-study bone complication in patients with NDMM [10]. Furthermore, median progression-free survival (a pre-specified exploratory endpoint) was 10.7 months longer with denosumab than with zoledronic acid on top of anti-myeloma therapy (*p* = 0.036), which may suggest an anti-myeloma effect of RANKL inhibition [10]. As denosumab is a relatively new therapy approved for use in this patient population, the long-term use of this treatment is of importance. A post hoc landmark analysis demonstrated superiority of denosumab over zoledronic acid for time to first on-study bone complication starting at 15 months (1 year after most bone complications; *p* = 0.039) [10]. However, given that denosumab is a relatively new therapy approved for use in patients in MM and has an alternative mode of action to zoledronic acid, the continuing need for large scale phase 3 trials is warranted.

We recognise that our approach has limitations. The reported incidence of bone complications could be influenced by differences in the sensitivities of imaging modalities used for their detection in different European countries. For example, computed tomography and positron emission tomography/computed tomography are more sensitive than plain radiographs [48–50]. The true burden of bone complications may be underestimated because only those that were symptomatic were likely to have been recorded. Additionally, the CRF did not allow the total number of bone complications experienced by each patient to be reported; some bone complications recorded between diagnosis and initiation of first-line treatment may not have been new because PF and SCC were only recorded at diagnosis if they led to the diagnosis, and the CRF did not allow SB and RB to be recorded at diagnosis. Furthermore, RI was only recorded at initiation of first-line treatment, and at diagnosis if it led to the diagnosis. Definitions of RI severity used in this study were more stringent than those routinely employed, so rates of moderate relative to mild RI are likely to be lower in our investigation than in comparable studies. RI severity was not recorded at diagnosis and normal renal function was not formally defined on the CRF. Bisphosphonate and analgesic use was not recorded in the period before initiation of first-line treatment, the time at which bone complications most commonly occurred. Therefore, the effect of bisphosphonate treatment on the incidence of bone complications could not be evaluated reliably. More generally, the study population may not have been representative of all patients with MM, and the clinical center and physician sample may not have been representative of all physicians treating MM in an individual country. The size of each national sample was also relatively small. The sampling method used helped to minimize selection and information bias but would not have eliminated it completely. Finally, no formal statistical tests were employed to compare patient groups and no adjustment was made for confounding variables.

## Conclusions

This large European retrospective chart review found that bone pain was common at diagnosis in individuals with NDMM, and that many patients experienced a bone complication (most commonly PF) between diagnosis and disease progression following first-line treatment. Bone complications were frequently associated with hospitalization and higher rates of analgesic use, adding to the burden on patients and health services. Although most patients were treated with bisphosphonates, our findings suggest that use of anti-resorptive therapy for prevention of bone complications may be suboptimal, and individuals with RI may have received bisphosphonates because no alternative treatment was available at the time of our study.

## Data Availability

The data sets generated and analysed during the current study are not publicly available, but they are available from the corresponding author on reasonable request. This study has been previously published as an abstract [51].

## Abbreviations

CI: Confidence interval
CKD: Chronic kidney disease
CrCl: Creatinine clearance
CRF: Case report form
eGFR: Estimated glomerular filtration rate
EU5: European Union 5 (France, Germany, Italy, Spain and the UK)
MM: Multiple myeloma
NDMM: Newly diagnosed multiple myeloma
PF: Pathologic fracture
RANKL: Receptor activator of nuclear factor kappa-B ligand
RB: Radiation to bone
RI: Renal impairment
SB: Surgery to bone
SCC: Spinal cord compression
UK: United Kingdom
US: United States
